# Differences in the gut microbiota of the black-faced spoonbill (*Platalea minor*) across different regions in China

**DOI:** 10.3389/fmicb.2025.1551391

**Published:** 2025-06-05

**Authors:** Pengcheng Yang, Habib Bati, Erhui Feng, Jingyao Hu, Xian An, Rongzeng Tan, Xinyue Dou, Taoyue Chen, Yifan Tao, Shuqiang Liu, Liangliang Yang

**Affiliations:** ^1^Ecology and Nature Conservation Institute, Chinese Academy of Forestry, Beijing, China; ^2^School of Ecology and Nature Conservation, Beijing Forestry University, Beijing, China; ^3^Hainan Dongzhaigang National Nature Reserve Authority, Haikou, China; ^4^Key Laboratory of Forest Protection of National Forestry and Grassland Administration, Beijing, China

**Keywords:** *Platalea minor*, gut microbiota, 16S rRNA gene sequencing, microbial diversity, Hainan Island (South China)

## Abstract

The black-faced spoonbill (*Platalea minor*) is a critically endangered, first-class protected bird species in China, and its gut microbiota is thought to play a crucial role in the bird’s ecological adaptability. However, regional variations in the gut microbiota of this species remain poorly understood. We aimed to investigate how the gut microbiota of *P. minor* differs across distinct regions in China to identify key factors influencing its composition and function. Fecal samples from black-faced spoonbills were collected in four regions of China (Shenzhen, Dongfang, Danzhou, and Xinying). We performed high-throughput 16S rRNA gene sequencing and bioinformatics analyses to characterize the diversity and abundance of gut microbiota at various taxonomic levels (phylum and family). Across all regions, the gut microbiota of *P. minor* was dominated by the phyla Firmicutes and Proteobacteria. Nevertheless, significant regional differences in microbial composition and predicted functional pathways were observed. Notably, the Shenzhen population’s microbiota showed the highest diversity in pathways related to light and chemical energy utilization. In contrast, the Danzhou population’s microbiota exhibited a higher representation of pathways related to metabolism and cellular repair. These findings indicate that gut microbiota profiles differ substantially by region. Our results suggest that regional environmental and dietary factors shape the gut microbiota of *P. minor*, which in turn may influence the species’ ecological adaptation. This study provides new insights into the ecological adaptability of the black-faced spoonbill and offers a scientific basis for developing effective conservation and habitat management strategies for this endangered species.

## 1 Introduction

Understanding the ecological adaptability of endangered species is critical for their conservation and management. Gut microbiota, as dynamic and essential microbial communities residing in the gastrointestinal tract, play a pivotal role in nutrient metabolism, immune regulation, and host-environment interactions (Larroya-García et al., 2018; Argaw-Denboba et al., 2024). For migratory birds, gut microbiota can adapt to environmental changes and food variability, enabling species to overcome ecological challenges during migration and habitat shifts. Despite this adaptive potential, systematic studies on gut microbiota in endangered migratory species remain limited, particularly in identifying regional variations and their ecological significance.

Accumulating evidence indicates that the gut microbiota of birds is shaped by multiple interacting factors. Previous research suggests that genetic relatedness can establish a foundational microbial signature (Deschasaux et al., 2018; Rinninella et al., 2019). Closely related birds may share core microbial features, whereas distantly related species, even if they occupy similar habitats or share comparable diets, can exhibit markedly different gut microbiota structures and functions (Feng et al., 2022; Long et al., 2023). Environmental variables, including climatic conditions, pollution, and regional ecosystem dynamics, further modulate these microbial assemblages, as migratory birds alter their microbiota to cope with different habitats en route (Hong et al., 2021; Zhang et al., 2022). Diet composition also profoundly affects microbial community structure and function, shaping which taxa thrive in a given host. For instance, phytophagous birds often exhibit lower microbial diversity dominated by Proteobacteria and Firmicutes, reflecting a specialized adaptation to plant-based diets (Juan et al., 2021). Collectively, these studies underscore that avian gut microbiota is not a static trait but a dynamic system responding to a complex interplay of genetic background, environmental pressures, and dietary inputs.

Recently, research on gut microbiota has become an important way to understand the mechanisms of animal health and ecological adaptation. The gut microbiota play key roles in host nutrient metabolism (Valdes et al., 2018; Wang et al., 2020), immune regulation (Maslowski and Mackay, 2011; Kamada et al., 2013; Sun et al., 2022; Ding et al., 2024), disease defense (Campbell et al., 2023; Cipelli et al., 2023), and influences the adaptability and migration patterns of birds (Lewis et al., 2017) through food and complex interactions with the environment (Thie et al., 2022). However, the gut microbiota composition and functions in many endangered birds remain poorly understood, including the black-faced spoonbill (*Platalea minor*), a first-class protected species in China. With a global population estimated at fewer than 7,000 individuals (Hong Kong Bird Watching Society, 2024), *P. minor* is critically endangered and relies heavily on key stopover sites along the East Asian-Australian Flyway for wintering and resting (Jung et al., 2018). Hainan Island and its surrounding regions, situated at the midpoint of this migration route, are particularly vital habitats for this species (Bamford et al., 2008). Despite the ecological significance of these habitats, little is known about how regional environmental conditions and food resources influence the gut microbiota of *P. minor* and facilitate its adaptation to diverse ecological niches. Understanding these dynamics is crucial for developing effective conservation strategies. Therefore, this study systematically investigates the regional differences in gut microbiota of *P. minor* across distinct habitats, focusing on environmental and dietary factors. By comprehensively analyzing the composition, diversity, and functional profiles of the gut microbiota, this work provides new insights into the ecological adaptation mechanisms of *P. minor* and offers a scientific foundation for its conservation and habitat management.

In this study, we systematically characterized the gut microbiota of *P. minor* across four regions in China (Shenzhen, Dongfang, Danzhou, and Xinying) using high-throughput 16S rRNA sequencing. We hypothesize that: (1) the gut microbiota of *P. minor* will exhibit significant structural and functional variation across these regions; (2) regional environmental factors will distinctly shape the composition and diversity of these microbial communities; and (3) dietary differences tied to local resource availability will drive functional differentiation within the gut microbiota. By addressing these objectives, our study not only systematically examines the regional differences in the gut microbiota of *P. minor* but also provides a scientific basis for guiding habitat management and conservation initiatives. This work provides new insights into the ecological adaptation of *P. minor* and contributes to the development of effective conservation and management strategies for this critically endangered species.

## 2 Materials and methods

### 2.1 Sample collection

**FIGURE 1:**
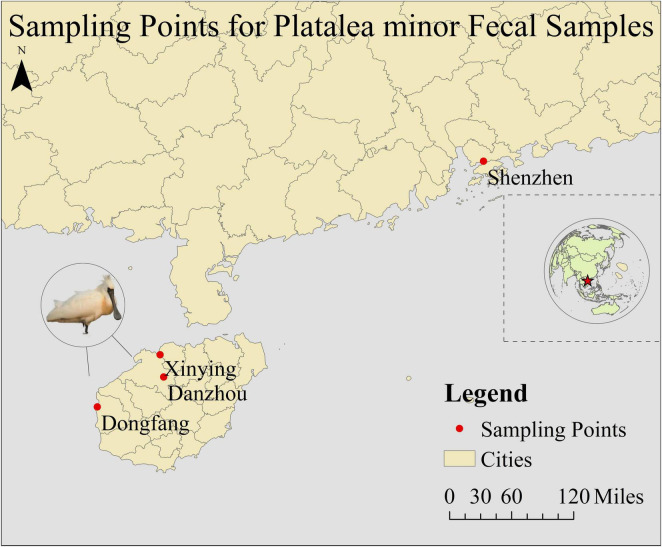
A map showing different sampling sites used in this study.

Fecal samples of *P. minor* were collected from November 2023 to April 2024 in Dongfang (17 samples), Danzhou (79), and Xinying (44) Cities in Hainan Province, and Shenzhen City in Guangdong Province (21), China (Figure 1). Detailed sample collection information is provided in Supplementary Table 1. We established contact with the conservation staff at four locations and requested that they collect as many fecal samples of black-faced spoonbills as possible during their patrols, with at least 20 samples to be collected at each sampling site. However, due to the limited number of samples collected in Dongfang (17 samples), we randomly selected 10 fecal samples from each site for microbiome analysis. During sampling, we wore disposable gloves and dipped a disposable cotton swab into the solid part of the stool. The swabs were placed in a disposable plastic bag, sealed, and marked with the sampling date, location, and bird species. All the cotton swabs and plastic bags used were sterilized with UV light, but due to the unavoidable exposure to environmental bacteria during field sampling, some contamination from outdoor bacteria may have occurred. Samples were stored at –20°C immediately after collection.

### 2.2 DNA extraction, 16S-rRNA gene polymerase chain reaction amplification, and sequencing

Fecal samples were prepared, and total DNA was extracted using the QIAamp DNA Stool Mini Kit (QIAGEN, Hilden, Germany) according to the manufacturer’s instructions. The DNA integrity was assessed on a 1.0% agarose gel containing ethidium bromide, and the DNA concentration was measured with a Qubit dsDNA HS Assay Kit (Life Technologies, Carlsbad, CA, USA). Extracted DNA was stored at –80°C. To monitor potential contamination, negative PCR controls (using sterile water in place of template DNA) were included during the amplification step, and no product was detected from them.

A one-step PCR was performed to amplify the V3–V4 region of the bacterial 16S rRNA gene using the primers 338F (5′-ACTCCTACGGGAGGCAGCA-3′) and 806R (5′-GGACTACHVGGGTWTCTAAT-3′). Each primer was tailed with an Illumina-specific index sequence to enable multiplexing. The PCR reaction (total volume: 10 μL) comprised 5–50 ng of template DNA, 0.3 μL each of 10 μM primers, 5 μL KOD FX Neo Buffer, 2 μL dNTPs (2 mM each), 0.2 μL KOD FX Neo, and ddH2O to a final volume of 10 μL. The thermal cycling conditions were: an initial denaturation at 95°C for 5 min; 25 cycles of 95°C for 30 s, 50°C for 30 s, and 72°C for 40 s; followed by a final extension at 72°C for 7 min.

PCR products were verified by 1.8% agarose gel electrophoresis, then purified using Agencourt AMPure XP Beads (Beckman Coulter, Indianapolis, IN, United States), and quantified using the Qubit dsDNA HS Assay Kit with a Qubit 4.0 Fluorometer. Equal amounts of each indexed amplicon were pooled to form the sequencing library.

Library quality was assessed using a Qsep-400 system (BiOptic Inc.). The library was sequenced on an Illumina NovaSeq 6000 platform (Illumina, Santiago, CA, United States) using the NovaSeq 6000 SP Reagent Kit v1.5 (500 cycles). The instrument’s integrated base-calling software directly generated FASTQ files, which include both sequencing and quality information.

### 2.3 Statistical and bioinformatics analyses

According to quality of single nucleotide, quality filtering was performed on the raw reads using Trimmomatic v0.33 (Bolger et al., 2014) with fastp parameters (–Q –y –g –Y 10 –l 100 –b 150 –B 150 –adapter_fasta), and primer sequences were subsequently removed using Cutadapt (version 1.9.1) (Martin, 2011) under parameters allowing up to 20% mismatches and a minimum coverage of 80%. Reads with a length shorter than 200 bp or containing homopolymers > 8 bp were excluded. Subsequently, USEARCH (version 10) (Edgar, 2013) was used to assemble paired-end reads and remove chimeras (UCHIME, version 8.1) (Edgar et al., 2011), resulting in high-quality sequences for further analysis.

Denoising and ASV generation were performed using QIIME2 (version 2020.6) (Bolyen et al., 2019) with the DADA2 (Callahan et al., 2016) plugin. Quality-controlled reads were denoised—merging paired-end reads and removing chimeric sequences—to yield the final set of non-chimeric reads. ASVs representing less than 0.005% of the total sequences were filtered out, and the remaining ASVs were normalized (rarefied) to account for relative abundance. Taxonomic annotation of the ASVs was performed using the Naïve Bayes classifier in QIIME2, referencing the SILVA database (release 138) (Quast et al., 2013) with a confidence threshold of 70%.

Alpha diversity indices (e.g., Chao1, Shannon) were calculated using QIIME2 and further visualized with box plots generated in R to compare microbial diversity across the four regional groups (Shenzhen, Dongfang, Danzhou, and Xinying). Pairwise statistical tests were performed to assess significant differences between groups.

**FIGURE 2:**
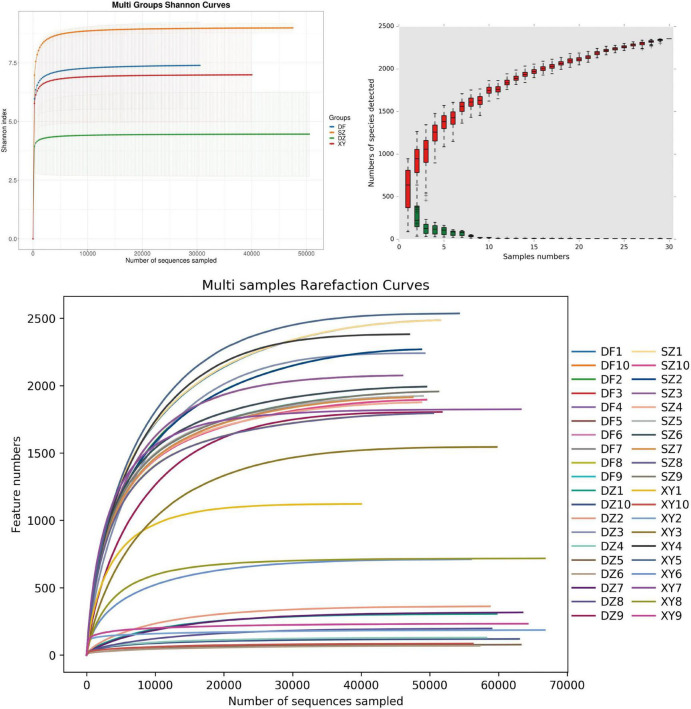
Coverage. **(A)** Rarefaction curve, **(B)** Shannon index, and **(C)** relative abundance accumulation curves. **(B)** The x-axis represents the sample size; the y-axis represents the number of species after sampling; the red boxplot components form the cumulative curve; the green boxplot components form the shared quantity curve.

In α-diversity analysis, rarefaction curves (Wang et al., 2012) are crucial tools for assessing whether the sequencing depth of a sample is sufficient. By randomly sampling sequences within each sample and plotting the relationship between the number of sequences and detected ASVs, the trend of microbial diversity in the sample with increasing sequencing depth can be intuitively displayed. If the rarefaction curve reached a plateau, the sequencing depth sufficiently captured most of the microbial diversity in the sample. The Shannon diversity index curve was used to assess the alpha diversity of the samples by calculating the Shannon index values and assessing its relationship with the sequencing depth, revealing the complexity and evenness of the microbial communities within the sample. The results from three analyses (Shannon index, species accumulation curve, and rarefaction curve) all indicated that the sampling effort and data were sufficient to capture the bacterial communities, yielding reliable analysis results (Figure 2). As shown in Figure 2B, the relative abundance accumulation curve displayed the cumulative abundance of different microbial species in the sample, illustrating that sufficient sequencing depth was achieved. Each red box represents the total number of species in a sample. Together, they form an accumulation curve that shows the rate at which new species appear with ongoing sampling—a steep rise indicates many new species, while a leveling-off suggests few additional species are found. Similarly, each green box indicates the number of common species in a sample; their collective curve reflects the appearance rate of common species.

We performed β diversity analyses to compare microbial community composition between samples using multiple distance-based metrics, including Bray-Curtis, Jaccard, and both weighted and unweighted UniFrac. These metrics quantify dissimilarity between samples, with higher values indicating greater differences. Dimensionality reduction methods, such as principal component analysis (PCA) (Dubois et al., 2010) and principal coordinates analysis (PCoA) (Gower, 1966) were used to project high-dimensional data into two- or three-dimensional spaces for visualization and interpretation, highlighting the differences between the groups. In addition, we constructed phylogenetic trees using the unweighted pair group method with arithmetic mean (UPGMA) in QIIME2 (version 2022.8). Specifically, UPGMA clustering was performed using binary Jaccard, Bray-Curtis, and unweighted UniFrac distance metrics to group samples iteratively based on their pairwise distances until a complete dendrogram was generated. Additionally, we applied both PERMANOVA and ANOSIM to evaluate significant differences in microbial community composition among the four regional groups: Shenzhen (SZ), Dongfang (DF), Danzhou (DZ), and Xinying (XY). PERMANOVA was used to determine how much of the overall variance in microbial community composition was explained by geographic grouping (global test), with *R*^2^ values indicating the proportion of variation attributed to regional differences. ANOSIM ranks distances and evaluates whether the average rank similarity within groups is greater than between groups. Subsequently, analysis was conducted using the vegan package in R language, and plotting was performed using Python, visualizing pairwise β-diversity distances between individual samples within and across regions.

In the visual analysis, ternary plots were used to show the relative abundances of the three major microbial taxa within the samples. We selected Dongfang (DF), Danzhou (DZ), and Xinying (XY) for this analysis because these locations are all within Hainan Island, allowing us to examine regional microbial community relationships within a geographically continuous area. Shenzhen (SZ) was excluded from the ternary plot because it is located across the Qiongzhou Strait from Hainan, making its microbial composition potentially influenced by distinct ecological factors. Taxa distribution bar plots displayed the abundance of major microbial species in samples from different regions, while species abundance clustering heatmaps employed color gradients and hierarchical clustering to illustrate similarities among samples. We used Linear Discriminant Analysis Effect Size (LEfSe) (Segata et al., 2011) to identify microbial taxa that differed significantly among the experimental groups. LEfSe applies a non-parametric test to detect differentially abundant taxa and then uses linear discriminant analysis (LDA) to evaluate effect sizes. To further explore co-occurrence patterns among microbial species, we performed Spearman’s rank correlation analysis on the species abundance profiles of all samples to identify co-occurrence patterns among species. Because no environmental measurements were collected, this analysis focused exclusively on species-to-species relationships. Correlations with coefficient (corr) > 0.1 and *p* < 0.05 were considered significant. We then visualized these significant correlations as a co-occurrence network using the igraph package (v2.1.4) in R (version 4.2.2), where nodes represented microbial taxa and edges denoted significant positive or negative correlations.

Finally, we conducted a functional analysis to compare the metabolic pathway activities among different groups of samples (e.g., Shenzhen, Dongfang, Danzhou, and Xinying). Specifically, PICRUSt2 (Douglas et al., 2019) was used to predict the functional potential of the microbial communities from the 16S rRNA gene data by aligning feature sequences against the IMG reference database, calculating the nearest sequenced taxon index (NSTI), and inferring gene family abundances. Predicted gene functions were then annotated using both the KEGG (Kanehisa and Goto, 2000) and the COG (Tatusov et al., 1997) database, allowing us to identify KEGG pathways and COG categories that differed significantly between groups. Additionally, we employed BugBase (v0.1.0) (Ward et al., 2017) to predict organism-level phenotypes. For BugBase analysis, although the platform historically requires an OTU-like input format, the input data in this study were derived from amplicon sequence variants (ASVs). Specifically, the normalized ASV table was mapped to the Greengenes database (version 13.5) (DeSantis et al., 2006) to generate an OTU-formatted table compatible with BugBase. This table was subsequently used to predict phenotypic traits (e.g., aerobic, anaerobic, pathogenic potential) and to estimate their relative abundance across samples. To further explore potential ecological functions, particularly those linked to biogeochemical cycles such as carbon, nitrogen, and sulfur cycling, FAPROTAX (Louca et al., 2016) was applied to map specific taxa to predicted ecological functions based on published, validated culture-based literature.

## 3 Results

### 3.1 Composition and structure of the gut microbiota in *P. minor* in different regions

#### 3.1.1 Analysis of differences in the α-diversity index

**FIGURE 3:**
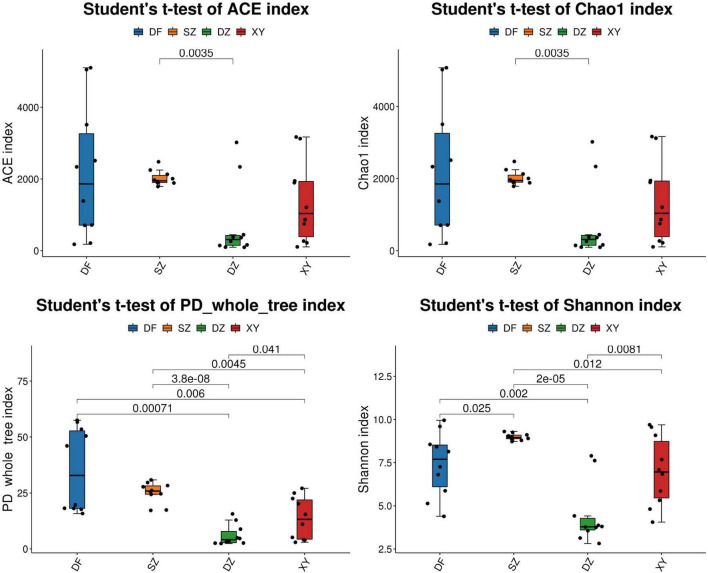
The differences in α-diversity index in the different regions. SZ, Shenzhen; DF, Dongfang; DZ, Danzhou; and XY, Xinying; PD, phylogenetic diversity; ACE, abundance-based coverage estimator. **(A)** ACE, **(B)** Chao1, **(C)** PD whole tree, and **(D)** Shannon.

The α-diversity index showed significant differences in the gut microbiota of *P. minor* across different locations. Specifically, significant differences were observed between Dongfang and Danzhou (Shannon, *p* = 0.002; PD_whole_tree, *p* = 0.0007), between Shenzhen and Danzhou (ACE, *p* = 0.0035; Chao1, *p* = 0.0035), between Danzhou and Xingying (Shannon, *p* = 0.0081), and between Shenzhen and Xingyi (PD_whole_tree, *p* = 0.0045) (Figure 3). The statistical significance of alpha diversity indices is summarized in Supplementary Table 2.

#### 3.1.2 Beta diversity analysis

##### 3.1.2.1 Analysis of composition differences

**FIGURE 4:**
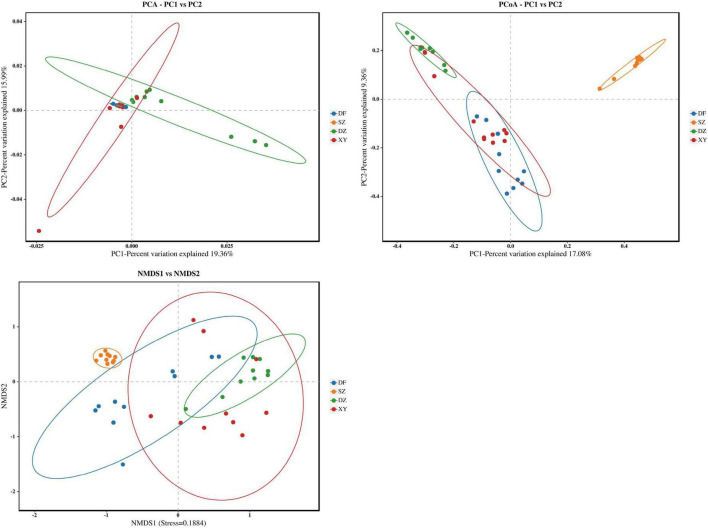
Results of the compositional difference analysis among the different regions. **(A)** PCA, **(B)** PCoA, and **(C)** NMDS. The oval circle indicates that it is a 95% confidence ellipse.

PCA analysis showed that the samples from the four regions were separated, and the aggregation of samples from Danzhou and Xinying showed differences in the gut microbiota of *P. minor* in the different regions. According to the PCoA analysis, the gut microbiota of *P. minor* in Shenzhen differed from that of the three Hainan Island regions. Non-metric multidimensional scaling (NMDS) analysis showed that the gut microbiota of *P. minor* varied between Shenzhen and Hainan, and that of *P. minor* in Xinying and Danzhou clustered closely together in the NMDS plot (Figure 4).

##### 3.1.2.2 Group reliability analysis

###### 3.1.2.2.1 UPGMA analysis

**FIGURE 5:**
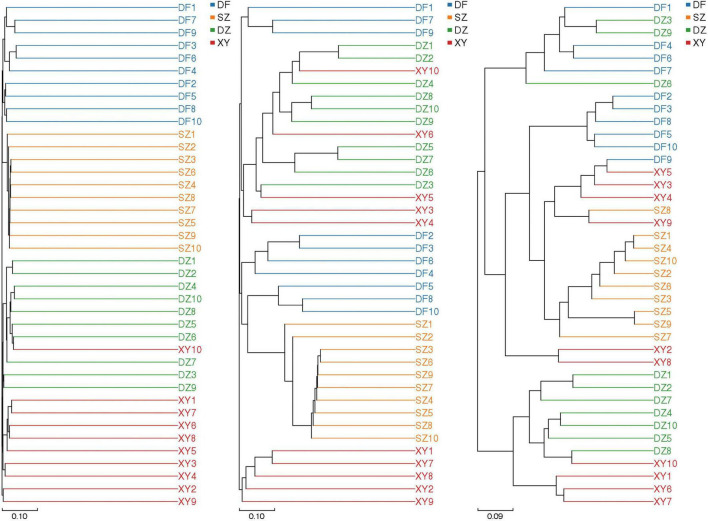
Phylogenetic trees showing the unweighted pair group method with arithmetic mean (UPGMA) analysis results for *P. minor*. **(A)** Binary Jaccard, **(B)** Bray Curtis, and **(C)** Unweighted Unifrac.

UPGMA analysis (Figure 5) revealed different clustering patterns depending on the metric used. Binary Jaccard (Figure 5A) revealed that the gut microbiota of *P. minor* in Dongfang (located in western Hainan) differed more from those in Xinying and Danzhou (located in northwest Hainan) than from those in Shenzhen (located across the Qiongzhou Strait from Hainan). However, Bray-Curtis (Figure 5B) and Unweighted UniFrac (Figure 5C) metrics indicated substantial overlap among samples from different locations. This suggests that while presence/absence-based metrics show regional separation, overall community composition and phylogenetic relationships are more intermixed.

###### 3.1.2.2.2 PERMANOVA and ANOSIM

**FIGURE 6:**
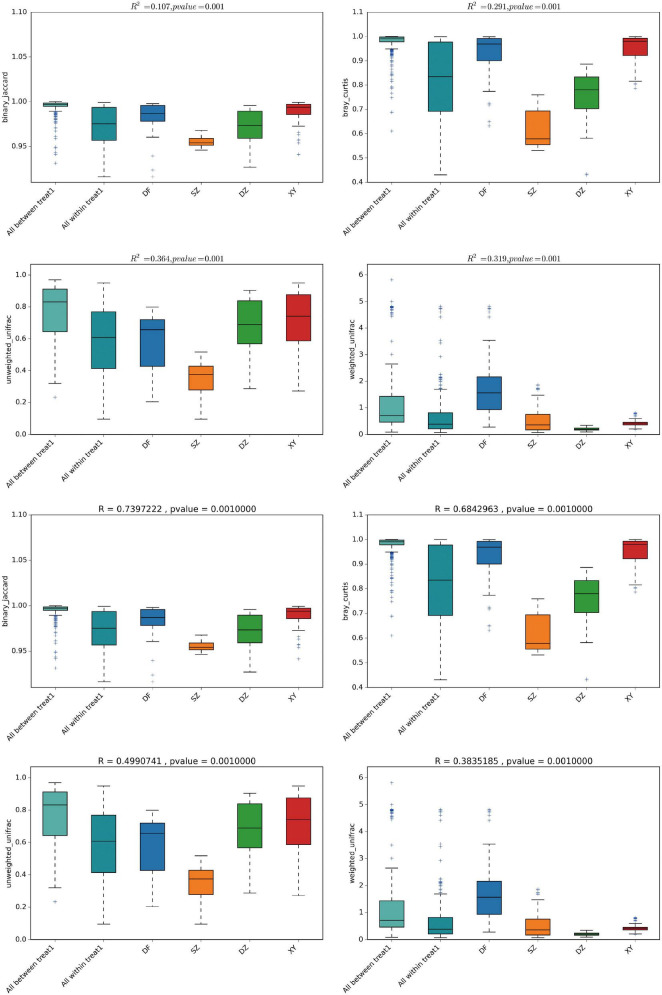
Box plots showing the PERMANOVA and ANOSIM results. **(A)** PERMANOVA and **(B)** ANOSIM. The four metrics used were: (a/a′) Binary Jaccard, (b/b′) Bray-Curtis, (c/c′) Unweighted UniFrac, and (d/d′) Weighted UniFrac.

PERMANOVA indicated significant gut microbiota differences among the four regions (*P* < 0.001; Figure 6A). In particular, Shenzhen’s microbiota differed from that of the three Hainan regions. This was consistent across all four distance metrics: (a) Binary Jaccard (*R*^2^ = 0.1070, *p* = 0.001), (b) Bray-Curtis (*R*^2^ = 0.2914, *p* = 0.001), (c) Unweighted UniFrac (*R*^2^ = 0.3637, *p* = 0.001), and (d) Weighted UniFrac (*R*^2^ = 0.3191, *p* = 0.001).

ANOSIM showed that the differences among the gut microbiota of *P. minor* in the four regions were greater than those within the groups, with high statistical confidence (Figure 6B), using four metrics: (a′) Binary Jaccard, *R* = 0.7397, *p* = 0.001; (b′) Bray-Curtis, *R* = 0.6843, *p* = 0.001; (c′) Unweighted UniFrac, *R* = 0.4991, *p* = 0.001; and (d′) Weighted UniFrac, *R* = 0.3835, *p* = 0.001.

PERMANOVA confirmed that geography explains a significant portion of gut microbiota variation (*P* < 0.001), with subtle differences among the three Hainan regions and a more pronounced difference between Shenzhen and Hainan. ANOSIM results supported this pattern.

#### 3.1.3 Taxa annotation and taxonomic analysis

**FIGURE 7:**
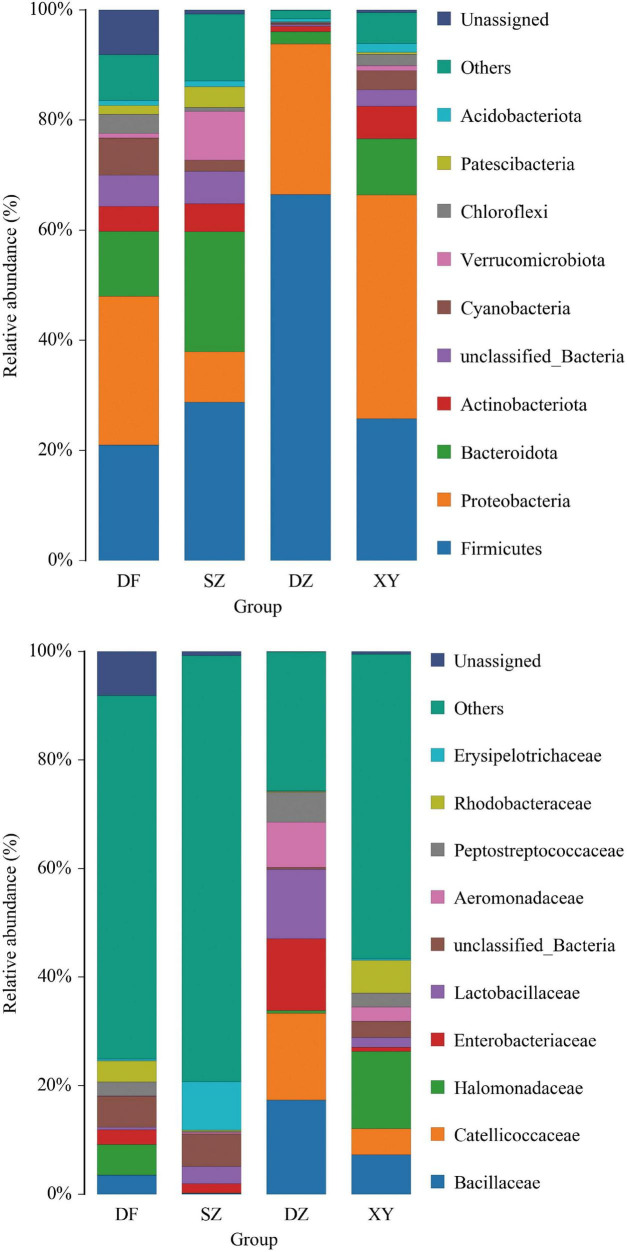
Bar graphs showing the taxa annotation. **(A)** Phylum and **(B)** Family levels. The category “Others” includes all bacterial families with relative abundances ranking outside the top 10, grouped together for visualization clarity. The category “Unassigned” represents sequences that could not be taxonomically classified at the family level based on the applied reference database.

The taxa bar chart at the phylum level (Figure 7A) shows that the gut microbiota of *P. minor* from the four regions was dominated by the phyla Firmicutes and Proteobacteria, consistent with common profiles in avian gut microbiota. Notably, the proportion of Firmicutes in the Danzhou group was unusually high, which may be related to local agricultural activity (Chang et al., 2016; Xu et al., 2024). Previous studies have shown that amphibians exposed to farmland environments tend to exhibit a higher Firmicutes-to-Bacteroidetes ratio, potentially enhancing energy absorption due to shifts in dietary composition. In the Shenzhen group, Firmicutes was also dominant, followed by Bacteroidetes and Proteobacteria. In contrast, Proteobacteria was the most abundant phylum in the Xinying group, and in the Dongfang group, Firmicutes and Proteobacteria were relatively balanced in abundance.

At the family level, as shown in Figure 7B, “Others” and “Unassigned” constituted a large proportion of the bacterial communities. In Shenzhen, “Others” and “Unassigned” accounted for 80% of bacteria, with *Erysipelotrichaceae* and *Lactobacillaceae* being the major identified families. Danzhou showed a more diverse family profile: unknown bacteria 30%, and families like Enterobacteriaceae, *Lactobacillaceae*, and *Aeromonadaceae* were prominent. Xinying had higher proportions of *Halomonadaceae*, *Bacillaceae*, *Rhodobacteraceae*, and *Catellococcaceae*; 70% were unassigned, and the remaining families were relatively evenly distributed.

#### 3.1.4 Cluster heatmap of species abundance

**FIGURE 8:**
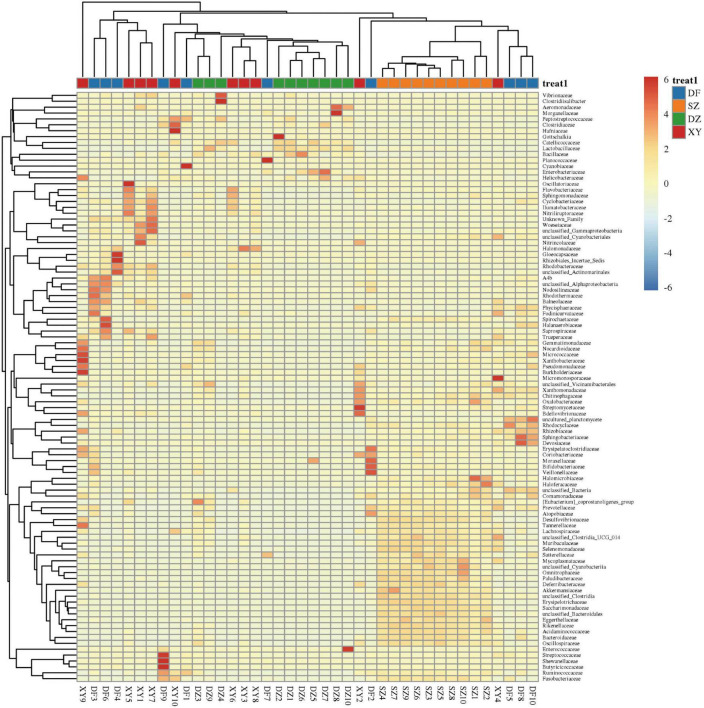
Heatmap showing the cluster analysis results of the gut microbiota of *P. minor*.

The heatmap shown in Figure 8 illustrates variations in the abundance and distribution of gut microbiota in fecal samples from different regions at the family level. Distinct clustering patterns suggest differences in microbial composition among sampling locations. Specifically, Danzhou (DZ) samples exhibit a higher relative abundance of *Catellicoccaceae*, *Lactobacillaceae*, and *Enterobacteriaceae*, while Shenzhen (SZ) samples show a greater presence of *Omnitrophaceae*, *Paludibacteraceae* and unclassified_*Cyanobacteriia*. Xinying (XY) samples show a greater presence of *Nitrincolaceae* and *Halomonadaceae*. Several low-abundance families are scattered across the samples but do not show a consistent regional pattern (Figure 8). The species abundance clustering heatmap showed that the gut microbiota composition of *P. minor* within the same region was similar, validating the geographic grouping.

#### 3.1.5 Microbial community differences (ternary diagram, LEfSe results) and correlation analysis

**FIGURE 9:**
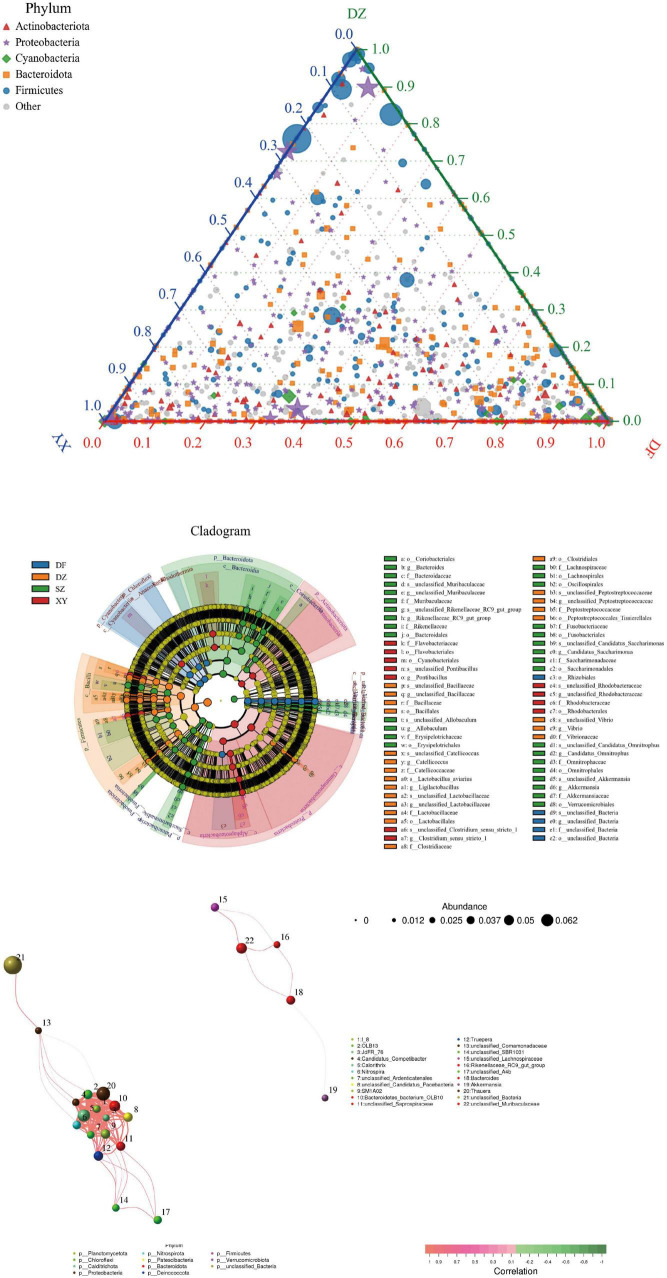
**(A)** Ternary phase diagram showing relative dominance of major phyla in Hainan regions (DF, DZ, XY). **(B)** LEfSe cladogram highlighting taxa with significant differences. **(C)** Co-occurrence network (Spearman correlation) among gut bacterial species. **(A)** Ternary phase diagram. The category “Others” includes all bacterial phylums with the average relative abundance outside the top 5. **(B)** LEfSe. The evolutionary branch diagram radiates from the inside out, with circles representing classification levels from phylum to species; each small circle at different classification levels represents a classification at that level, and the size of the small circle is directly proportional to the relative abundance; the coloring principle is to uniformly color species without significant differences in yellow, and other species with differences are colored according to the group with the highest abundance of that species. Different colors represent different groups, and nodes of different colors indicate microbial communities that play an important role in the group represented by that color. The *p*-value of species with significant differences is in the table of Supplementary material. **(C)** Species correlation analysis. Nodes represent individual bacterial species, with larger nodes indicating higher average abundance. Edges (lines) represent significant correlations, with thicker edges indicating stronger relationships. Red edges indicate positive correlations, while green edges indicate negative correlations.

The ternary phase diagram (Figure 9A) illustrates the relative composition and distribution of the dominant bacterial phyla among the gut microbiota of *P. minor* from the three Hainan Island regions: Danzhou (DZ), Dongfang (DF), and Xinying (XY). In the diagram, each vertex represents one of the three regions, and bacterial taxa are positioned based on their relative abundances in these locations. The results indicate that: *Proteobacteria* and *Firmicutes* were most dominant in Danzhou; *Actinobacteriota* and *Proteobacteria* were enriched in Dongfang; *Bacteroidetes* and “Others” (includes all bacterial phylums with the average relative abundance outside the top 5) were relatively more abundant in Xinying. This visualization highlights the regional variability in gut microbiota composition, showing that different bacterial phyla are predominant in different sampling locations. Danzhou had high relative abundances of *Firmicutes* and *Cyanobacteria*, suggesting a diverse community; Dongfang showed moderate diversity; Xinying had higher *Bacteroidetes* and “Others,” indicating an even more diverse community. The Danzhou and Dongfang groups show some similarities in gut microbiota, as did the Danzhou and Xinying groups, whereas the Dongfang and Xinying groups displayed more distinct differences.

The LEfSe results show significant differences in microbial taxa in fecal samples of *P. minor* in the four regions at the species level (e.g., k__Bacteria.p__Bacteroidota.c__Bacteroidia, LDA_scores = 4.9791020331, *p* = 0.00002845), and identify groups with significant differences in abundance. Danzhou showed differences mainly in *Firmicutes* (especially order *Bacillales*); Xinying had multiple differential taxa in *Proteobacteria* and *Bacteroidetes*; Dongfang had many in *Bacteroidetes* and *Firmicutes*; Shenzhen had several in *Bacteroidetes* and *Firmicutes* (Figure 9B). The LDA values corresponding to Figure 9B are shown in Supplementary Table 3.

The species correlation analysis plot (Figure 9C) was constructed using Spearman’s rank correlation (corr > 0.1, *p* < 0.05, corr represents the correlation between two nodes, with a higher value indicating a stronger correlation) to identify potential co-occurrence patterns among bacterial species in the gut microbiota of *P. minor*. The network analysis revealed a core set of species with strong positive correlations, including *Candidatus*_*Competibacter*, *Nitrospira*, and *Bacteroides*, which formed highly interconnected clusters. Additionally, some species, such as unclassified_*Muribaculaceae* and unclassified_*Lachnospiraceae*, were found to be more isolated, exhibiting fewer or weaker correlations with other taxa.

#### 3.1.6 Functional analysis

##### 3.1.6.1 KEGG pathway differential analysis by PICRUSt2

**FIGURE 10:**
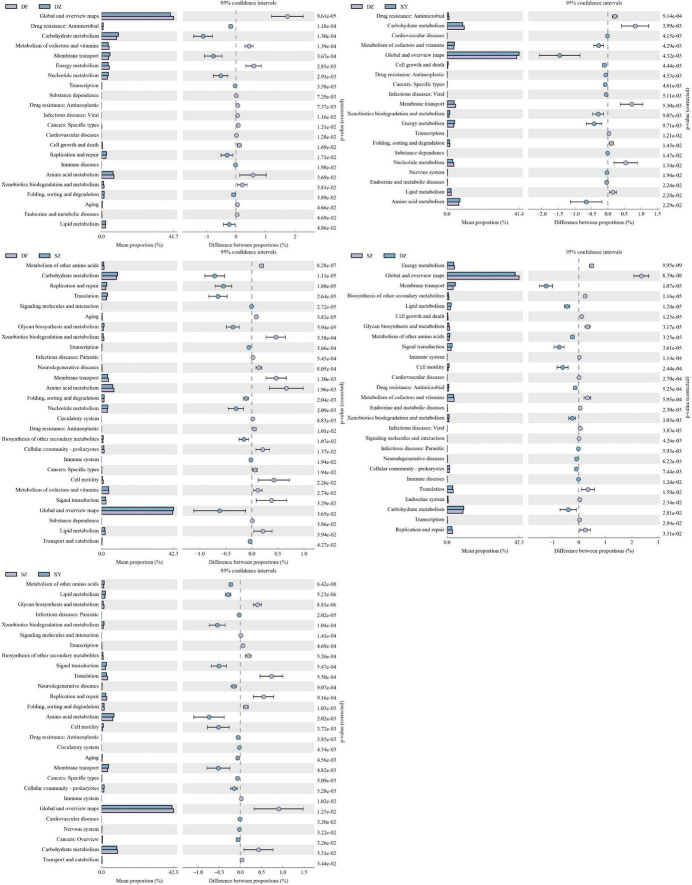
Results of the differential analysis of KEGG metabolic pathways. **(A)** DZ vs. DF, **(B)** DZ vs. XY, **(C)** SZ vs. DF, **(D)** SZ vs. DZ, and **(E)** SZ vs. XY. Dongfang and Xinying did not have significant differences, so no figure was provided.

KEGG pathway differential analysis results showed significant differences between Shenzhen and Dongfang and Xinying in Metabolism of other amino acids (SZ vs. DF, *p* = 0.000000828; SZ vs. XY, *p* = 0.0000000642), between Shenzhen and Dongfang in Carbohydrate metabolism (SZ vs. DF, *p* = 0.0000111), and in Lipid metabolism when compared to Danzhou, and Xinying (SZ vs. DZ, *p* = 0.0000124; SZ vs. XY, *p* = 0.00000523). Additionally, Shenzhen and Danzhou exhibited significant differences in Energy metabolism (SZ vs. DZ, *p* = 0.00000000995) and Cell growth and death (*p* = 0.0000125). Differences between Danzhou and Dongfang were observed in Carbohydrate metabolism (DZ vs. DF, *p* = 0.00013) and Metabolism of cofactors and vitamins (*p* = 0.000139), whereas Danzhou and Xinying showed significant differences in Energy metabolism (DZ vs. XY, *p* = 0.00971), Metabolism of cofactors and vitamins (*p* = 0.00429), and Cell growth and death (*p* = 0.00444) (Figure 10). Finally, Dongfang and Xinying did not have significant differences (*p* > 0.05), so no figure was provided. Functional annotation using COG is listed in Supplementary Table 4.

##### 3.1.6.2 Statistical map of COG functional classification by PICRUSt2

**FIGURE 11:**
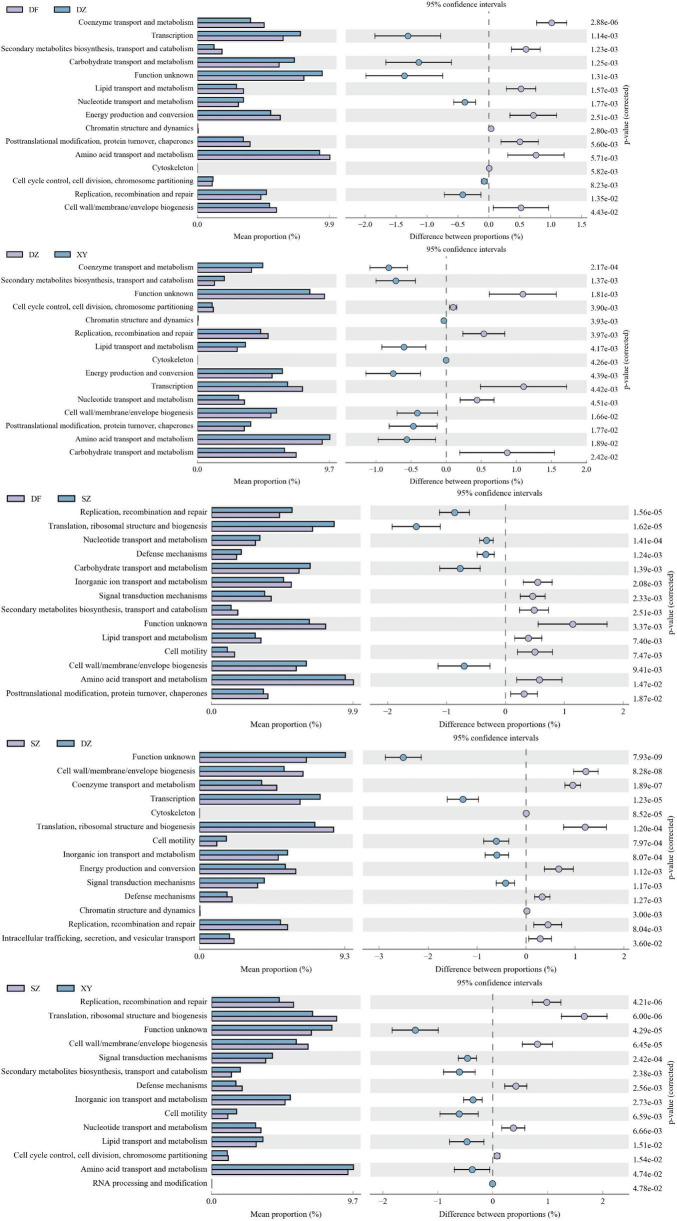
Statistical plot showing the difference in various Cluster of Orthologous Genes (COG) functions of the gut microbiota of *P. minor* among the four regions. **(A)** DF vs. DZ, **(B)** DZ vs. XY, **(C)** DF vs. SZ, **(D)** SZ vs. DZ, and **(E)** SZ vs. XY. Dongfang and Xinying did not have significant differences, so no figure was provided.

Significant differences in various COG functions of the gut microbiota of *P. minor* were observed among the four regions. The Danzhou and Dongfang groups exhibited significant differences in Coenzyme transport and metabolism (DZ vs. DF, *p* = 0.00000288), Nucleotide transport and metabolism (*p* = 0.00177), Energy production and conversion (*p* = 0.00251), and Transcription (*p* = 0.00114). The Danzhou and Xinying groups differed significantly in Coenzyme transport and metabolism (DZ vs. XY, *p* = 0.00000288) and Cell cycle control, cell division, and chromosome partitioning (*p* = 0.00039), and significant differences in Transcription (DZ vs. SZ, *p* = 0.0000123) were observed between the Danzhou and Shenzhen groups. The Dongfang and Shenzhen groups exhibited significant differences in Carbohydrate transport and metabolism (DF vs. SZ, *p* = 0.00139), Replication, recombination and repair (*p* = 0.000156), Signal transduction mechanisms (*p* = 0.00233) and Translation, ribosomal structure and biogenesis (*p* = 0.0000162). The Shenzhen and Xinying groups exhibited significant differences in Replication, recombination and repair (SZ vs. XY, *p* = 0.00000421), Signal transduction mechanisms (*p* = 0.000242) and Amino acid transport and metabolism (*p* = 0.0474) (Figure 11). Finally, Dongfang and Xinying did not have significant differences (*p* > 0.05), so no figure was provided.

##### 3.1.6.3 BugBase phenotype prediction plot

**FIGURE 12:**
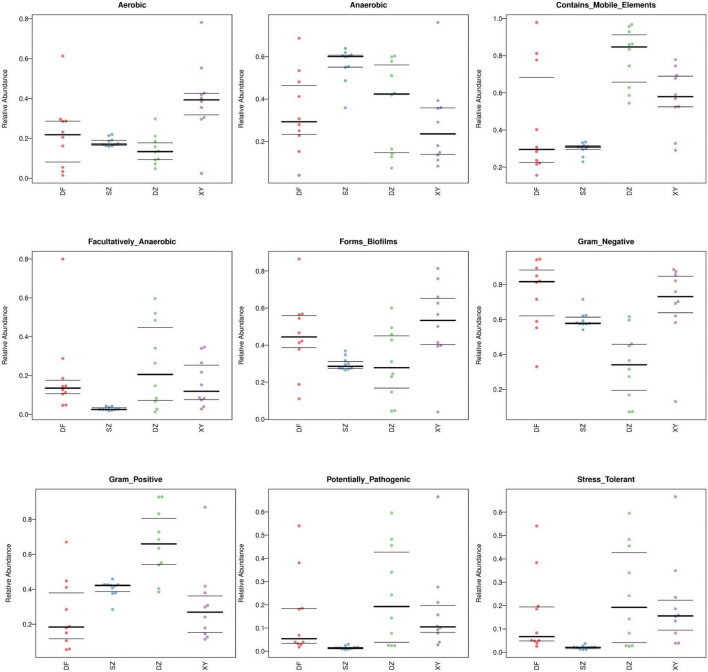
BugBase phenotype prediction plot showing the difference in microbial community composition and function among the different regions. **(A)** Aerobic, **(B)** anaerobic, **(C)** contains mobile elements, **(D)** facultatively anaerobic, **(E)** forms biofilms, **(F)** gram negative, **(G)** gram positive, **(H)** potentially pathogenic, and **(I)** oxidative stress tolerant.

The BugBase phenotypic prediction analysis (Figure 12) revealed significant differences in microbial functional traits across the four regions (Shenzhen, Dongfang, Danzhou, and Xinying). These findings suggest that regional differences in microbial phenotypes may be influenced by differences in host diet and gut environment. Differences were observed in predicted microbial oxygen tolerance, Gram staining characteristics, and biofilm formation capacity, among other traits. Specifically, (A) Aerobic, *p* = 0.00289; (B) Anaerobic, *p* = 0.00805; (C) Contains Mobile Elements, *p* = 0.000470; (D) Facultatively Anaerobic, *p* = 0.000684; (E) Forms Biofilms, *p* = 0.0171; (F) Gram Negative, *p* = 0.000557; (G) Gram Positive, *p* = 0.000557; (H) Potentially Pathogenic, *p* = 0.000184; (I) Oxidative Stress Tolerant, *p* = 0.000134. Taking “Aerobic” as an example, the *p*-values between each group were: DF vs. SZ = 0.529, DF vs. DZ = 0.472, DF vs. XY = 0.0230, SZ vs. DZ = 0.0786, SZ vs. XY = 0.00627, DZ vs. XY = 0.00627. All results are detailed in Supplementary Table 5.

##### 3.1.6.4 Faprotax function prediction

**FIGURE 13:**
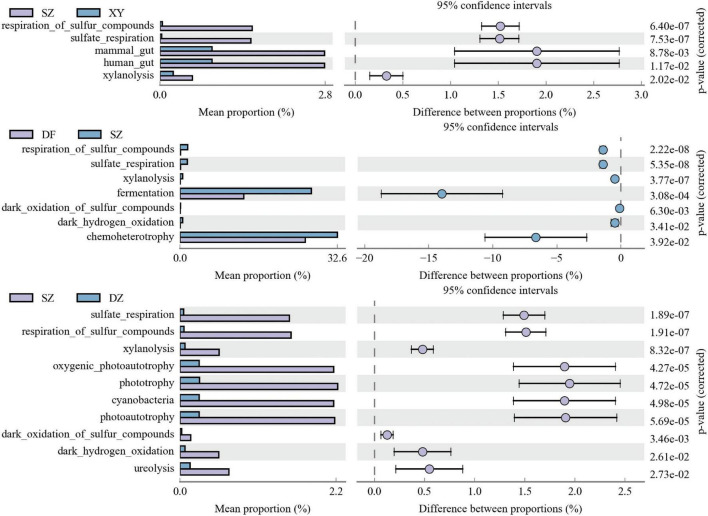
Faprotax function prediction plots of the gut microbiota of *P. minor* across the different regions. **(A)** SZ vs. XY, **(B)** DF vs. SZ, and **(C)** SZ vs. DZ.

There were no significant differences in the Faprotax functional prediction results of the gut microbiota of *P. minor* among Danzhou (DZ), Dongfang (DF), and Xinying (XY) in Hainan Island (*p* > 0.05), hence figures were not provided. The Faprotax functional prediction results showed significant differences in the gut microbiota of *P. minor* between Shenzhen (SZ) and other regions (Figure 13). In Shenzhen, the microbiota exhibits higher mammal gut (SZ vs. XY, *p* = 0.00878), human gut (*p* = 0.0117), sulfur compound respiration (SZ vs. XY, sulfate respiration, *p* = 0.000000753 and dark oxidation of sulfur compounds, *p* = 0.000000640; SZ vs. DF, sulfate respiration, *p* = 0.000000189 and dark oxidation of sulfur compounds, *p* = 0.00346), fermentation (SZ vs. DF, *p* = 0.000308), chemoheterotrophy (*p* = 0.0392), and higher phototrophic activity (SZ vs. DZ, phototrophy, *p* = 0.0000472 and oxygenic photoautotrophy, *p* = 0.0000427). FAPROTAX functional predictions are summarized in Supplementary Table 6.

## 4 Discussion

The global population of *P. minor* is estimated to be only 6,988 (Hong Kong Bird Watching Society, 2024). Because of the difficulty in collecting samples from this species, research on their gut microbiota remains limited. To our knowledge, this is the first study to use 16S rRNA Illumina MiSeq high-throughput sequencing technology to compare the gut microbiota of *P. minor* across different regions. We analyzed the diversity of the microbiota and conducted differential analyses at the phylum and family levels. These analyses provide deeper insights into the functional potential of *P. minor* during migration, offering valuable scientific data for understanding microbial ecology and informing conservation efforts for this species. We analyzed the gut microbiota composition and functional potential of *P. minor* across different geographic regions to better understand microbial community variation in this species.

### 4.1 Similarity across regions attributed to core microbiota

The gut microbiota composition of *P. minor* varied among the four regions, yet core microbial phyla such as *Firmicutes*, *Proteobacteria*, *Bacteroidetes*, and *Actinobacteria* consistently dominated across all sampled regions. This is consistent with the findings for gastrointestinal microbiota in other bird species (Waite and Taylor, 2015; Hird, 2017).

Although the overall gut microbiota composition of *P. minor* varied across Shenzhen, Dongfang, Danzhou, and Xinying, a consistent core microbiota—defined as microbial taxa shared across all individuals—was observed in all populations. The species correlation analysis indicated that *Candidatus*_*Competibacter*, *Nitrospira*, and *Bacteroides* occupied important positions within the core gut microbiota. Previous studies reported that *Candidatus*_*Competibacter* is associated with acetate degradation (Shi et al., 2023) and glycogen accumulation (Izadi et al., 2020), Nitrospira participates in nitrification within the nitrogen cycle (Daims and Wagner, 2018; Chang et al., 2023), and Bacteroides contributes to carbohydrate breakdown and short-chain fatty acids (Ríos-Covián et al., 2016), especially propionate (Facchin et al., 2024). Therefore, these microbial groups may play important roles in food digestion and nutrient metabolism of *P. minor*. The relatively abundant but taxonomically unclassified bacteria (e.g., unclassified_*Bacteria*, unclassified_*Muribaculaceae*, unclassified_*Lachnospiraceae*) may interact with the core microbiota to support essential gut functions. While the precise roles of these unclassified taxa in *P. minor* remain unclear, previous studies have shown that functionally related members of these bacterial families contribute to nutrient metabolism (Wang et al., 2023b), immune regulation (Kim et al., 2024), and intestinal barrier protection (Rios-Covian et al., 2017). Among them, unclassified_*Muribaculaceae* has been associated with butyrate and tryptophan metabolism, providing anti-inflammatory effects (Tang et al., 2024) and maintaining gut barrier function (Wang et al., 2023a), while unclassified_*Lachnospiraceae* affects host energy balance (Delzenne and Cani, 2011), blood glucose control (Vacca et al., 2020), and insulin sensitivity (Koh et al., 2016). It may also produce short-chain fatty acids (Louis and Flint, 2017) such as butyrate (Tremaroli and Bäckhed, 2012), which help maintain gut health (Topping and Clifton, 2001), modulate immunity, and reduce inflammation (Overby and Ferguson, 2021). These functions are key to stabilizing the gut ecosystem and supporting host health. Studies have shown that even after long migrations, the core gut microbiota of migratory birds, such as swan geese, remains highly conserved (Wu et al., 2018). Therefore, the core microbiota likely play a vital role in maintaining gut health, regulating immunity, and managing energy metabolism during *P. minor* migration, a phenomenon broadly applicable across bird species.

### 4.2 Influence of geographical location

Alpha diversity analysis (Shannon index) showed that microbial richness was highest in Shenzhen, followed by Dongfang, then Xinying, and lowest in Danzhou. This suggests that geographical location played a significant role in shaping the gut microbiota composition. A study involving nearly 200 fecal samples from Kirtland’s warblers found significant differences in bacterial species between samples collected in Michigan and the Bahamas, with the same individual exhibiting different gut microbiota compositions depending on the location (Skeen et al., 2021). This highlights the close relationship between gut microbiota composition and the geographical location. Simultaneously, there were clear differences in the diversity and richness of the gut microbiota between different regions. Microbial diversity was higher in Shenzhen and Xinying (more complex communities), whereas Dongfang and Danzhou were dominated by certain taxa, showing distinct regional characteristics. These results demonstrate significant differences between the gut microbiota of *P. minor* from Shenzhen and Hainan Island.

Significant differences in the gut microbiota of *P. minor* were also observed across different regions of Hainan Island, where the sampling locations include Dongfang (DF), Danzhou (DZ), and Xinying (XY). The ternary plot further indicated that the DF and DZ groups show some similarities in gut microbiota, as did the DZ and XY groups, whereas the DF and XY groups displayed more distinct differences. Beta diversity analysis confirmed this, showing that the gut microbiota of the DZ and XY groups were more closely clustered, possibly because of their geographical proximity, resulting in a more consistent microbiota composition. Geographic differences are the key factors influencing microbiota variation. DF, on the southern coast of Hainan, is rich in tropical rainforests and coastal wetlands, whereas DZ has vast farmland and abundant water resources. Additionally, the Limu Mountain range creates hills and terrain that separate DF and DZ. This topographical separation potentially leads to different climate and ecology in each area, which may influence *P. minor*’s migration routes and habitat selection, and subsequently alter their gut microbiota. According to the phylum-level community composition, the differences in gut microbiota in DZ may also be linked to the region’s agricultural activities (such as the use of pesticides and fertilizers), which may have altered the food sources of farmland wildlife (such as farmland frogs) and increased the incidence of harmful pathogens in farmland habitats (Köberl et al., 2011; Hartmann and Six, 2022).

Functional prediction based on FAPROTAX and BugBase analyses revealed regional variation in microbial metabolic potential. In *P. minor* from Shenzhen, microbial communities exhibited enriched phototrophic, fermentative, and chemoheterotrophic activities, potentially reflecting adaptation to specific local conditions. Excluding Shenzhen (non-Hainan site), Dongfang had the lowest relative abundance of certain bacterial groups among the Hainan locations. This may indicate a better health status for *P. minor* in Dongfang, with less exposure to external oxidative stresses (e.g., antibiotics, pesticides) (Feng and Wang, 2020) and a more stable anaerobic gut environment. In contrast, Danzhou samples were enriched in protein metabolism and cell repair functions, whereas Xinying samples showed higher abundance of functions related to cell cycle regulation and DNA repair. These patterns suggest region-specific microbial responses to environmental stressors. A study on geladas (*Theropithecus gelada*) showed that the gut microbiota composition changes with precipitation and temperature variations, and although the overall influence of temperature on the gut microbiota is relatively small, it has been found that in colder seasons, bacterial communities involved in energy, amino acid, and fat metabolism increase in abundance (Baniel et al., 2021). This suggests that when temperature regulation and nutritional stress occur simultaneously, gut fermentation activity is stimulated, helping species maintain their energy balance during challenging periods. This finding is consistent with seasonal changes in the gut microbiome observed in a study on great tits (*Parus major*) (Liukkonen et al., 2024), further supporting the critical role of environmental factors in regulating the gut microbiome. These findings highlight the potential role of environmental factors in shaping gut microbial function, and may provide a useful framework for understanding geographic variation in the microbiota of *P. minor*.

### 4.3 Potential dietary influence on gut microbiota composition and function

Functional profiles differed across regions, and some of these differences may be associated with variation in diet. For instance, previous sections highlighted that *P. minor* in Shenzhen showed distinct metabolic activities. The results of the Beta diversity analysis indicate that the gut microbiota of *P. minor* in Shenzhen significant differences compared to three locations in Hainan Island. Specifically, the gut microbiota diversity in Shenzhen was the highest, with several taxa belonging to *Bacteroidetes* and *Firmicutes*. While diet has been shown to influence gut microbiota composition in birds (Wang et al., 2021), further investigation is required to determine the specific dietary factors contributing to these regional differences in *P. minor*. Food sources from different habitats can significantly affect bird gut microbiota. For example, studies have shown that exogenously increasing the concentration of toxic substances such as nicotine and neonicotinoids in the diet of the Orange-tufted Sunbird (*Cinnyris osea*) significantly increases the number of nicotine and neonicotinoid-degrading bacteria in the gut microbiota (Gunasekaran et al., 2020), indicating the crucial role of diet in shaping gut microbiota composition. While diet is known to influence gut microbiota composition and function in birds (Kohl, 2012), our study did not include direct dietary data to confirm the relationship between food availability and microbiota function in *P. minor*. Future studies integrating detailed dietary analysis will be crucial to clarify how local food sources contribute to functional differentiation of gut microbiota in *P. minor*.

In summary, our study demonstrated significant regional differences in both the composition and predicted functional profiles of the gut microbiota in *P. minor*. At the phylum level, *Firmicutes* and *Proteobacteria* were dominant across all regions, but their relative abundances varied substantially. For instance, *Firmicutes* was enriched in Danzhou and Shenzhen, while *Proteobacteria* was more prevalent in Xinying. At the family level, notable differences were observed in *Enterobacteriaceae*, *Lactobacillaceae*, and *Halomonadaceae*, which showed region-specific enrichment patterns, suggesting localized microbial adaptation. Functionally, predicted microbial activities such as energy metabolism, oxidative stress tolerance, and DNA repair varied among regions. These functions may reflect the microbiota’s ecological role in helping hosts respond to environmental stressors, which is particularly relevant for migratory and endangered species like *P. minor*. The observed variation may be linked to differences in habitat quality, anthropogenic disturbance, or food resource availability across sampling sites. Our findings provide valuable baseline data for the microbial ecology of *P. minor* and underscore the potential of gut microbiota analysis as a complementary tool in conservation planning. Monitoring changes in microbial community composition and function could support assessments of habitat quality, population health, or adaptive capacity under environmental change. Future research incorporating dietary profiling, environmental metrics, and host genomic data will be essential to further elucidate the ecological and evolutionary significance of gut microbiota in this species.

## Data Availability

The raw sequence data generated in this study have been deposited in the NCBI Sequence Read Archive (SRA) under BioProject accession number PRJNA1262093. The data are publicly available at: https://www.ncbi.nlm.nih.gov/bioproject/1262093.

## Supplementary material

Supplementary Table 1 Sample collection record table (sampling locations, dates, and sample IDs).

Supplementary Table 2 *P*-values for alpha diversity comparisons among regions.

Supplementary Table 3 LDA values and group associations from LEfSe species analysis (corresponding to Figure 9B).

Supplementary Table 4 Functional annotation summary based on COG (Clusters of Orthologous Groups).

Supplementary Table 5 BugBase phenotype prediction data matrix.

Supplementary Table 6 FAPROTAX function prediction results table.
