# Correction: Differences in the gut microbiota of the black-faced spoonbill (*Platalea minor*) across different regions in China

**DOI:** 10.3389/fmicb.2025.1682719

**Published:** 2025-08-29

**Authors:** Pengcheng Yang, Habib Bati, Erhui Feng, Jingyao Hu, Xian An, Rongzeng Tan, Xinyue Dou, Taoyue Chen, Yifan Tao, Shuqiang Liu, Liangliang Yang

**Affiliations:** ^1^Ecology and Nature Conservation Institute, Chinese Academy of Forestry, Beijing, China; ^2^School of Ecology and Nature Conservation, Beijing Forestry University, Beijing, China; ^3^Hainan Dongzhaigang National Nature Reserve Authority, Haikou, China; ^4^Key Laboratory of Forest Protection of National Forestry and Grassland Administration, Beijing, China

**Keywords:** *Platalea minor*, gut microbiota, 16S rRNA gene sequencing, microbial diversity, Hainan Island (South China)

In the published article, there was an error in affiliation 1. Instead of “Key Laboratory of Forest Protection of National Forestry and Grassland Administration, Research Institute of Forest Ecology, Environment and Protection, Chinese Academy of Forestry, Beijing, China”, it should be “Ecology and Nature Conservation Institute, Chinese Academy of Forestry, Beijing, China”.

In the published article, there was an error regarding the affiliation for Liangliang Yang. As well as having affiliation 1 they should also have affiliation “Key Laboratory of Forest Protection of National Forestry and Grassland Administration, Beijing, China.”

The original version of the article has been updated.

